# Antimicrobial Resistance in Diverse Ecological Niches—One Health Perspective and Food Safety

**DOI:** 10.3390/antibiotics14050443

**Published:** 2025-04-28

**Authors:** Nedjeljko Karabasil, Milica Mirković, Ivan Vićić, Ivana Perić, Nevena Zlatković, Bojana Luković, Ina Gajić

**Affiliations:** 1 Department of Food Hygiene and Technology, Faculty of Veterinary Medicine, University of Belgrade, Bulevar Oslobodjenja 18, 11000 Belgrade, Serbia; nedja@vet.bg.ac.rs; 2 Department of Industrial Microbiology, Faculty of Agriculture, University of Belgrade, Nemanjina 6, 11000 Belgrade, Serbia; petrusicm@agrif.bg.ac.rs (M.M.); ivana.peric@agrif.bg.ac.rs (I.P.); 3 Department of Plant Diseases, The Institute for Plant Protection and Environment, Teodora Drajzera 9, 11000 Belgrade, Serbia; nevena_blagojevic@yahoo.com; 4 College of Health Science, Academy of Applied Studies, Cara Dusana 254, 11080 Belgrade, Serbia; bojanall@yahoo.com; 5 Institute for Microbiology and Immunology, Faculty of Medicine, University of Belgrade, dr Subotića 8, 11000 Belgrade, Serbia; ina.gajic@med.bg.ac.rs

**Keywords:** bacteria, pathogens, susceptibility, environment, animal, human, food

## Abstract

Antimicrobial resistance (AMR) is a multi-sectoral, systemic, and global issue worldwide. Antimicrobial use (AMU) is a key factor in the selection of resistant bacteria within different ecological niches, from agriculture to food-producing animals to humans. There is a question regarding the extent to which the use of antibiotics in livestock production and the primary food production sector influences the selection and transmission of resistant bacteria and/or resistant genes throughout the food chain and thus contributes to the complexity in the development of AMR in humans. Although the trends in the prevalence of foodborne pathogens have changed over time, the burden of ecological niches with resistance genes, primarily in commensal microorganisms, is of concern. The implementation of the harmonized surveillance of AMU and AMR would provide comprehensive insights into the actual status of resistance and further interventions leading to its reduction. Tracking AMR in different ecological niches by applying advanced genome-based techniques and developing shared AMR data repositories would strengthen the One Health concept.

## 1. Introduction

In recent years, antimicrobial resistance (AMR) has posed a significant challenge worldwide, affecting diverse ecological niches—from agriculture to human health—leading to economic losses and, ultimately, death [1,2,3,4,5]. The main driver for the emergence of AMR in bacteria is uncontrolled antimicrobial use (AMU), particularly in human health care and hospitals [6]. Recently, increasing attention has been paid to the AMU in food-producing animals as a significant source of environmental burden and the possible transmission of resistance to humans [7,8,9]. Possible transmission routes for the entry of resistant bacteria into the human population from animal sources are through direct contact, indirectly through food, or from the environment through water [10,11]. However, the exact pattern of transmission is still under investigation. While AMU has been identified as the leading cause of resistance, several factors may contribute to its development and further spread, including global demographic changes, importation, poor hygiene and the development of opportunistic infections, and the excretion of antimicrobial residues into the environment via manure [12,13]. The additional use of antimicrobial and chemical agents in preventing plant diseases through agricultural production [14] makes the AMR issue even more complex, requiring a multi-system and multi-sectorial approach through a One Health concept.

This study addresses the current state of AMR in different niches, from agriculture through food-producing animals to humans, and future perspectives on reducing AMR.

## 2. Agriculture and Environment

The presence and spread of antibiotics are widespread in various ecological niches, which has led to antibiotic resistance in bacteria in agriculture and the environment [15]. Given the constant increase in the human population and the need for food, agricultural production is often forced to find new pathways that will ensure high yields and safety.

### 2.1. Antibiotic Resistance in Agriculture

Various plant diseases can lead to considerable losses in agricultural production worldwide, estimated at billions of dollars. A particular challenge is the control of bacterial diseases where bacteria colonize healthy plant tissue, multiply rapidly and infect tissue. Copper-based compounds are most commonly used to control bacterial diseases, but can only be used in the pre-flowering phase. They may inactivate the pathogens before they penetrate the host tissue, as the pathogen is otherwise protected from the toxic effects of the chemicals by the surrounding tissue. In years with favorable weather conditions for disease development, which include high humidity and appropriate temperatures, bacterial diseases can lead to complete crop destruction. In this context, growers often resort to the uncontrolled use of copper preparations, which induces pathogen resistance [16].

In agriculture, five antibiotics are most commonly reported for use: streptomycin, oxytetracycline, kasugamycin, oxolinic acid, and gentamicin [14]. The beginning of the use of antibiotics in agriculture is closely linked to fire blight (FB) of pome fruit, a disease caused by phytopathogenic bacteria *Erwinia amylovora*. Known for more than 100 years, the disease was first described in the United States and spread across North America, New Zealand, the Middle East, and Europe [17]. The first use of antibiotics in agriculture, particularly streptomycin, was in the 1950s in the USA to control FB in commercial orchards [18].

As a result of the numerous treatments in pome fruit orchards, antibiotic-resistant bacteria (ARB) first appeared in several locations in the western United States in the 1970s [19]. In particular, streptomycin is most commonly used to control *Pseudomonas syringae*, the causal agent of blossom and fruit infection of apple and pear fruit; *Xanthomonas campestris*, the causal agent of bacterial spot disease of tomatoes and peppers; *Xanthomonas arboricola* on fruit and nuts; and *Agrobacterium tumefaciens*, the causal agent of crown gall on roses [18,19,20]. It is authorized for minor use to control bacterial diseases on potato tubers, tobacco seedlings, and other vegetable seedlings both in the field and in the greenhouse [17,21]. There are two mechanisms for resistance to streptomycin in the FB pathogen samples. The most common is the spontaneous mutation of the chromosomal gene *rpsL*, which is responsible for the production of the ribosomal protein, and the second is acquired resistance, when the pathogen acquires plasmids carrying the tandem gene pair *strA* and *strB*, which encodes the production of an aminoglycoside phosphotransferase that inactivates streptomycin [17].

In the following years, oxytetracycline (OTC) was the antibiotic of choice in orchards [22]. In fact, OTC was the first antibiotic approved as a pesticide for use in pear and peach orchards in 1974 [18]. The occurrence of Huanglongbing disease (HLB) in citrus orchards, which is caused by the non-culturable bacterium “*Candidatus* Liberibacter asiaticus”, is considered the most destructive citrus disease since its appearance in the Americas [23,24]. The application of OTC using different methods (sprays, injection, boot injection) is used to suppress the disease [18,25,26]. OTC-resistant strains are rarely reported. To date, no *E. amylovora* strains with resistance to moderate concentrations of OTC have been detected [17]. OTC-resistant strains have been reported in *P. syringae*, *X. a. pv. Pruni*, and *A. tumefaciens* [14], where various genes could be responsible for tetracycline resistance—the expression of efflux pumps and the production of enzymes that inactivate the antibiotic and ribosomal protein [14,17,27].

Kasugamycin is an aminoglycoside that was first authorized for use in agriculture in 2018, mainly against streptomycin-resistant phytopathogenic bacteria to control fire blight [28], as well as for pome fruit, cherries, and walnuts [18]. It is also used in Japan and some Asian countries to control bacterial and fungal diseases in rice [18,19]. The presence of the aac(2′)-IIa gene in two bacterial rice pathogens, *B. glumae* and *Acidovorax avenae*, has been shown to cause resistance to kasugamycin [19]. The resistance of *E. amylovora* to kasugamycin is induced in in vitro tests, but it has not been detected in field isolates. In addition, one study evaluated the potential for spontaneous resistance and found that a two-step mutation process is required. These mutants were significantly reduced in fitness [14,18].

Oxolinic acid (OA) has been used commercially in Israel since 1998, with success in the control of fire blight [29]. It is also used in Japan to control bacterial panicle blight in rice caused by *Burkholderia glumae* [17,30]. OA is not approved for use in agriculture in the United States, primarily due to the emergence of antibiotic resistance. The mechanism for the development of OA resistance is still unknown, but it is hypothesized that it may be due to chromosomal mutations rather than gene acquisition through horizontal gene transfer [31].

Gentamicin has been used in Chile and Central America to control vegetable diseases caused by *Pectobacterium*, *Ralstonia*, *Xanthomonas*, and *Pseudomonas* species. It is also used in Mexico to control vegetable crops as well as for the treatment of FB. Gentamicin is not approved for use in the United States due to its clinical significance [17,19,20]. Resistance to gentamicin was only reported in *Xanthomonas oryzae* pv. *oryzae*, when *aacA3* encodes enzyme that deactivates gentamicin [14].

Antibiotics such as ningnanmycin, validamycin, and zhongshengmycin are frequently used in China. This shows that other antibiotics could potentially be used in plant protection, but their use and efficacy are not yet monitored [14].

Doubtlessly, antibiotics as a tool for plant protection will remain valuable. Nowadays, it is very difficult to say reliably which countries use antibiotics. Some countries have well-defined guidelines about antibiotic usage, but unfortunately, most countries do not have established regulations or any database. Only the United States, New Zealand, and India publish information on the amount of antibiotics used on crops [14]. In the United States, antibiotics used in plants account for less than 0.5% of total antibiotic use [18,32]. In Serbia, antibiotics are not allowed for use in plant protection [33]. Nevertheless, their increased use has led to the emergence of resistant plant pathogenic bacterial strains in the past. Considering the trade in plant material and products, it is highly important to increase awareness about antibiotic use [18]. In this way, the amount of antibiotic use would reduce the presence of antibiotic residues as well as ARB in other ecological niches.

### 2.2. Presence of Antibiotics, Antibiotic-Resistant Bacteria, and Antibiotic-Resistant Genes in Water

Over the past two decades, several studies have reported the presence of ARB and antibiotic-resistant genes (ARGs) at different concentrations in water bodies in different parts of the world: Europe [34,35,36], Asia [37,38], and Australia [39]. Large amounts of antibiotics are released into the environment through human activities (clinical therapy, livestock and poultry farming, and pharmaceutical production) [37]. The main receptors and hotspots for the release of ARB into the environment are wastewater treatment plants, where a large number of antibiotics and a high density of ARB are present in wastewater [34,40,41]. Most interesting is wastewater from hospitals and farms, which are probably the main source of ARB and ARGs released into the environment [42]. Nowadays, several factors are accelerating the flourishing development of antibiotic resistance in various environmental media, including anthropogenic activities and the evolution of microorganisms to combat antibiotics [43,44].

In addition to wastewater, surface waters appear as both a habitat and a transport system for microorganisms and play a key role in this spread. Most antibiotics and ARGs present in surface waters come from wastewater from the previously mentioned sources of antibiotics (hospitals, farms, pharmacies) [36,37,45]. Multiple classes of antibiotics have been detected in drinking water systems in some European countries [46,47,48], Canada [49,50], China [37,51], and the USA [52,53]. Despite the use of wastewater treatment plants, antibiotics, ARB, and ARGs can still be present in high concentrations in downstream rivers [54]. Due to inadequate or incomplete treatment, many drinking water reservoirs are heavily contaminated with antibiotics and ARGs from upstream wastewater [55]. As a result, drinking water with antibiotics and ARGs represent a risk to drinking water safety [37]. Therefore, it is essential to improve systems and techniques in wastewater plants.

Bonetta et al. [34] examined the antibiotic resistance of influent and effluent samples of two drinking water treatment plants (DWTPs) and three wastewater treatment plants (WWTPs) located in the northwest of Italy. They consider WWTPs as a “hotspot” for the spread of antibiotic resistance, while water samples after treatment in DWTPs and WWTPs exhibited a significant reduction in ARB and ARGs, but still exhibited antibiotic-resistant bacteria and genes. These results highlighted the importance of the examination of antibiotic resistance in different samples of water, especially in the urban water cycle, as well as drinking water that presents a potential route of transmission to humans.

Surface waters can serve as a “marketplace” where different microorganisms (especially in the presence of antibiotics from wastewater) can acquire new resistances [56]. Antibiotic-resistant Gram-negative bacilli (e.g., *Enterobacteriaceae*, *Pseudomonadales*) are favored because many species are native to water bodies and enable a high level of cross-species genetic exchange [57]. In particular, bacteria that produce extended-spectrum beta-lactamases (ESBLs) have become ubiquitous over the last decade. They occur in clinical settings, in human communities, and in animals (wildlife, pets, and livestock) [58]. The excessive use of antimicrobial agents in veterinary medicine is one reason for the increase in ESBLs in animal populations. This leads to the occurrence of ARB in the animals themselves and to the contamination of food of animal origin, which contaminates the soil or surface water [35].

The Danube, as the second largest river in Europe, was examined for the resistance profile of *Escherichia coli* and *Klebsiella* spp., which were isolated from 68 sample sites along the course of the river. A total of 629 *E. coli* and 319 *Klebsiella* spp. were isolated, out of which 61 isolates of *E. coli* (9.7%) and 7 isolates of *Klebsiella* spp. (2.19%) had acquired resistance to more than three antibiotics. Furthermore, two *E. coli* and two *Klebsiella* spp. isolates were tested as positive on ESBL genes. Besides *E. coli* and *Klebsiella* spp., the authors also examined the presence of ESBL and carbapenamase harboring *Enterobacteriaceae* as examples of clinically relevant resistance mechanisms. They isolated 35 ESBL *Enterobacteriaceae*, 17 *E. coli*, 13 *Klebsiella pneumoniae*, and 5 *Enterobacter* spp. The obtained results showed that only five isolates were not classified as multiresistant, while nine isolates showed resistance to one antibiotic out of six representative classes. The study revealed the river Danube as reservoir of antibiotic-resistant *Enterobaceriaceae* [35].

The global distribution of carbapenemase-producing strains has been detected in rivers in different regions of the world [59]. In the study by Lepuschitz et al. [36], neither ESBL-producing nor carbapenem-resistant *K. pneumoniae* isolates were found in five water samples taken upstream of the cities of Vienna (Danube), Linz (n = 2, Danube and Traun), Klagenfurt (Glan), and Innsbruck (Inn). On the other hand, five samples taken downstream of the cities contained multiresistant *K. pneumoniae* isolates (Danube, Traun, Glan, and Inn). In addition, a comparison was made between isolates from water samples and 95 clinical *K. pneumoniae* isolates based on whole-genome sequencing, whereby three clusters were identified. Cluster 1 and cluster 2 consisted of isolates from water and clinical samples that were carbapenem-resistant strains. Cluster 3 consisted of three ESBL-producing strains isolated from a water sample and two clinical samples. The cities where the patient isolates of clusters 2 and 3 were collected matched the locations of the water samples downstream of these cities. The results of this study reveal clinically relevant strains in the environment, which is alarming and appears to be a future public health problem that requires heightened attention.

Source water contains a considerable number of bacteria, besides treated drinking water (finished water) and tap water, in Louisiana [40]. Several pure cultures were isolated and identified, and the bacteria that were consistently present in the water sample each month in the period from September 2014 to September 2015 were *E. coli*, *Enterobacter cloacae*, *K. pneumoniae*, *Staphylococcus warneri*, *Bacillus*, *Pseudomonas*, and *Enterococcus* spp. The bacteria were resistant to some of the common antibiotics such as oxacillin, clindamycin, vancomycin, erythromycin, neomycin, chloramphenicol, tetracycline, kanamycin, and streptomycin. However, the water treatment plant removed these bacteria from tap water, as well as from finished water.

China represents one of the world’s largest producers and consumers of antibiotics, among which 46.1% of antibiotics are used in the animal industry [60]. Most of these antibiotics end up in the environment via waste from animal husbandry. Plants can take up and bioaccumulate antibiotics from the environment, which endangers human health when consumed [61]. Besides animal industry and plants, drinking water sources in China can be polluted with antibiotics and ARGs. A study by Hu et al. [37] revealed the presence of antibiotics in frequencies above 70% in drinking water sources in East China, while concentrations of different antibiotics ranged from 19.68 to 497 ng/L. In addition, 41 ARG subtypes and four intergrase genes were investigated, while 18 ARG subtypes and the class I intergrase gene *int1* were detected in water samples. The obtained results indicate antibiotic resistance pollution in the drinking water sources of East China.

In a preliminary study carried out by Mohamad et al. [62], in three samples from DWTPs, multidrug-resistant *Enterobacteriaceae* and heterotrophic ARB were isolated in river water sources. The obtained results showed that, out of 56 isolates, 24 (40%) *E. coli* and *Salmonella* spp. were multidrug-resistant isolates.

Waste water, rivers, water drinking sources, and lakes also have the potential to store and accumulate ARGs due to the longer retention time of pollutants, as the water retains pollutants from wastewater that circulate slowly in the lakes [63]. In a study by Filipić et al. [64], ten samples from glacial lakes of the Western Balkans were examined for the presence of ARGs. The RND efflux pump genes were the most abundant in the metagenome of Lake Plav, followed by *ermB*, *bla_TEM_*, *aacA*, and *aadA* genes. Genes for RND efflux pumps and *ermB* were detected in the Black Lake metagenome, while all resistance genes tested were found in the Donje-Bare Lake metagenome, with the *bla_TEM_* gene being the most abundant. The western glacial lake sediments analyzed in this study differed in terms of exposure to the human population, chemical properties, and bacterial community composition. Since Plav is a relatively small town, the author assumed that the human population exerts little selection pressure through the use of antibiotics in medicine or agriculture.

Once in the environment, antibiotics used in agriculture, veterinary, or human medicine are linked to the contamination of water in different parts of the environment. Furthermore, the use of antibiotics consequently leads to the rise of ARB and ARGs and their transfer from one environment to another and, finally, to humans. In that sense, some effort must be made to reduce the possibility of ARB and ARGs entering and spreading into the environment. The most effective and direct approach may be the judicious use of antibiotics in health protection and agricultural production. In addition, new and effective wastewater treatment processes need to be developed to improve the efficiency of ARG removal in wastewater treatment plants. Furthermore, the feasibility of the agricultural application of sewage sludge or irrigation with treated wastewater needs to be thoroughly discussed, as ARGs can enter the soil and groundwater.

## 3. Food Production and Veterinary Public Health

Over the past decade, demands for high-quality animal protein have increased, posing a significant challenge to improving animal health and welfare, reducing morbidity and mortality in food-producing animals, and overall economic losses in the food industry [65,66]. The misuse or overuse of antimicrobial agents in animal husbandry leads to the emergence of AMR in pathogenic bacteria, the complexity of the development of genetic mechanisms in the present microbiome, and further selective pressure on commensal microorganisms [67,68].

### 3.1. Antimicrobial Use (AMU) in Food-Producing Animals

The AMU in intensive livestock production is rising, with the prediction that total antibiotic consumption in the farm system will increase by 104,079 tonnes by 2030 [69,70]. Although antibiotics are used for therapeutic purposes, it is evident that their application for non-therapeutic purposes, through prophylaxis, metaphylaxis, and as growth promoters, is still present [2,71]. Subtherapeutic AMU is influenced by several factors, such as inadequate drug selection, inappropriate dosing, non-bacterial infection, and inadequate treatment duration [65,72]. Through irrational, suboptimal doses of antibiotics in farm animals, ideal conditions for the development of AMR occur. Considering the large biomass of farm animals, the chances of mutations in microorganisms are high, leading to shifts in the gut microbiome, facilitating the presence and further spread of resistance microorganisms and their genes, not only to the antibiotics used but also against other antibiotics, influenced by complex mechanisms of co-resistance [73]. It has also been established that 75% of the antibiotics used in farm animals are excreted via feces and urine, affecting antibiotic residues’ release into the environment and selective pressure on the microbiome [74].

The strategy for mitigating AMR includes several steps: classifying antimicrobial drugs, monitoring AMU in farm animals, and the surveillance of pathogenic microorganisms for which animals serve as reservoirs [75,76]. In human and veterinary medicine, antimicrobial drugs have been classified by the World Health Organization (WHO) and World Organization for Animal Health (WOAH) according to their importance: critically important, highly important, and important. Based on the above approach, two classes of antibiotics are important for both sectors: third- and fourth-generation cephalosporins and fluoroquinolones [77]. Although the highest level of resistance in farm animals is toward tetracyclines, sulfonamides, and penicillins due to their availability and low cost [66], in recent years, there has been an increasing trend toward the emergence of resistance to critically important antibiotics. Another issue that arises is co-resistance. Resistance to carbapenems, which are banned in veterinary medicine, is most likely a consequence of using third- and fourth-generation cephalosporins. Therefore, Quebec has restricted the use of critically important antibiotics to human medicine in food-producing animals, including polymyxins, third- and fourth-generation cephalosporins, and fluoroquinolones [78]. Most developed countries have implemented a strategy for monitoring AMU in veterinary medicine (Table 1) [79,80]. However, a harmonized, quantitative methodological protocol has not yet been established, making it challenging to monitor AMU in livestock between different countries, let alone at the level of different ecological niches [81]. Future directions for surveillance should include monitoring AMU in different animal species, emphasizing developing publicly available databases similar to those in human medicine. Reducing AMU in food-producing animals has yielded some results, lowering the occurrence of resistant pathogens among animals, with limited prospects for reducing resistance in humans [82]. The burden of the ecological niche with resistance genes, primarily in commensal microorganisms, as well as the mechanisms of resistance transmission could lead to complexity in reducing the AMR. Therefore, the reduction in AMU should have a multi-sector approach, not limited to food-producing animals, with mandatory susceptibility testing before using antimicrobial drugs.

### 3.2. Surveillance of AMR in Food Production

During all stages of its production, food can be contaminated with various microorganisms, which, under certain conditions, can lead to foodborne infections in humans or spoilage. The most commonly investigated pathogens in food are *Salmonella*, *Campylobacter* spp., Shiga toxin-producing *E. coli*, *Listeria monocytogenes*, and *Staphylococcus aureus* [83]. Trends in the prevalence of specific pathogens in food have changed over time, especially in developed countries, due to the implementation of preventive measures. Due to the low prevalence of *Salmonella* spp., monitoring *E. coli* is recommended as an indicator of commensal microorganisms for the easier tracking of resistance genes present at the microbiome level, considering Gram-negative bacteria. *Enterococcus faecalis* and *Enterococcus faecium* are recommended as indicator microorganisms for monitoring the resistance of Gram-positive bacteria [84]. Detecting genes resistant to the last-resort antibiotics used in human medicine, carbapenems and colistin (polymyxin E), is of concern. Resistance genes to carbapenems (*bla_VIM-1_*, *bla_NDM-1_*, and *bla_NDM-5_*) and colistin (*mcr-1* to *mcr-9*) have been identified in isolated *Salmonella* from pigs, poultry, and humans, indicating a multi-sector pattern of resistance and a threat to public health [85].

The surveillance of antimicrobial resistance in pathogenic microorganisms involves the application of classical microbiological and molecular techniques. The standard approach includes culture and susceptibility testing, phenotypic confirmatory tests, and the determination of the minimum inhibitory concentration [65,86,87,88]. Advanced molecular techniques for the detection of resistance genes, mutation recognition, the identification of resistance determinants, and the analysis of resistomes have a high level of discrimination even among very similar bacteria species [65,89,90]. Genome-based approaches should be used to identify and relate AMR mechanisms from food-producing animals, environments, and humans [85,90]. Likewise, the WHO recommends using whole-genome sequencing (WGS) as a tool to strengthen foodborne disease surveillance and response, including its application in outbreak investigations [91]. Publicly available tools such as the Comprehensive Antibiotic Resistance Database (CARD) and ResFinder facilitate the interpretation of WGS data by linking resistance genes to corresponding phenotypic traits, thereby supporting more accurate AMR surveillance across the One Health spectrum. Whole-genome long-read sequencing is, to date, the most accurate genotyping method, which allows for AMR determinants mediated by chromosomal point mutations and resistance linked to mobile genetic elements to be distinguished [84]. WGS allows for the identification of resistant genes and mutations and the prediction of gene transmission between different bacterial species, which, together with phylogenetic analysis, provides a deeper insight into the pattern of AMR between different regions and countries [92]. Thus, a cross-sectional study investigating AMR at the human–animal–environment interface found genetically closely related strains of *Campylobacter jejuni* and *Salmonella enterica* in chicken manure, retail chicken meat, and the feces of asymptomatic farm workers. Notably, the majority of *Salmonella* isolates were multidrug-resistant (*S. enterica* serovar Muenchen), harboring the emerging *S. infantis* pESI megaplasmid, which confers resistance to multiple antibiotics. These findings underscore the transmission of MDR foodborne pathogens from animals to humans through the food chain [93]. Metagenomics, as a DNA-based approach, is the next step in monitoring AMR, which directly analyzes genetic material from the entire sample, allowing AMR patterns to be recognized across the entire diversity of present microorganisms [94].

## 4. Public Health and One Health Approach

When it comes to food, AMR presents a dual problem: (i) it is a source of pathogenic bacteria that, if resistant, can make treatment challenging when infection requires antibiotic use; (ii) food also serves as a reservoir for both pathogenic and non-pathogenic bacteria, which can carry resistance genes. These genes can be transferred to the normal microbiome of the digestive tract and pose an issue, as they may be passed on to future pathogenic bacteria, compromising their treatment.

### 4.1. Pathogenic Microorganisms and Foodborne Infections

#### 4.1.1. Overview of Foodborne Pathogens and AMR

Every year, unsafe food leads to 600 million cases of foodborne illnesses and 420,000 deaths worldwide. The WHO estimates that 33 million years of healthy life are lost globally annually due to unsafe food consumption, a figure that is likely an underestimation [95]. Each year, there are 1.7 billion cases of childhood diarrhea worldwide, often linked to contaminated food and water, leading to 525,000 deaths [96].

Most cases of infectious diarrhea in immunocompetent persons are self-limiting, typically caused by viruses, and require only symptomatic care. Bacterial acute diarrhea is also usually self-limiting, with antimicrobial treatment generally not recommended. However, antimicrobials are indicated for patients with persistent fever, bloody or persistent diarrhea, severe illness that restricts mobility, and HIV-positive or immunocompromised individuals. AMR is an increasing threat to the effective treatment of bacterial infections causing diarrhea, driven by the widespread use of antibiotics in both food animal production and clinical settings.

#### 4.1.2. Common Zoonotic Foodborne Pathogens

The most common zoonotic bacterial pathogens responsible for foodborne infections, along with the number of confirmed cases, hospitalizations, and deaths according to the European Food Safety Authority and the European Centre for Disease Prevention and Control, are displayed in Table 2 [97].

##### Non-Typhoidal *Salmonella*

The WHO lists non-typhoidal *Salmonella* (NTS) as one of the major global causes of diarrheal disease, with over 2500 NTS serotypes or serovars [98]. In 2023, the European Union (EU) reported 16.9% zoonotic salmonellosis cases per 100,000 population, making it the second most common foodborne infection after campylobacteriosis. The top three serotypes, *S.* Enteritidis, *S.* Typhimurium, and its monophasic variant (1,4,[5],12:i:-), accounted for over 70% of human cases, continuing a trend since 2014. In 2023, these serovars caused 84.8% of cases, while *S.* Infantis remained the fourth most common serovar. Isolates were mainly linked to broiler sources, followed by pigs, laying hens, turkeys, and cattle [99]. The FoodNet report for 2019 shows that *Salmonella* infection ranked second in the United States of America (USA) after *Campylobacter*, with 14.2% of cases per 100,000 population. For over a decade, five *Salmonella* serotypes (Enteritidis, Newport, Typhimurium, Javiana, and *S*. 1,4,[5],12:i:- have been predominant. Over 75% of human salmonellosis in the USA was attributed to seven food categories: chicken, fruits, pork, seeded vegetables (e.g., tomatoes), other produce (e.g., fungi, herbs, nuts, root vegetables), turkey, and eggs [100]. NTS infection occurs when bacteria bypass the stomach’s acid barrier and invade intestinal cells, causing inflammation. If the infection spreads, it can lead to invasive NTS (iNTS), especially in those with a weakened immune system and newborns. Septicemia is the most common complication and the overall pooled case–fatality ratio of iNTS is estimated at 17.1% in Africa, 14.0% in Asia, 9.9% in Europe, and 9.6% in the Americas [101]. Health authorities are concerned about the growing cases of gastroenteritis and sepsis caused by NTS strains resistant or multidrug-resistant (MDR) to common antibiotics like beta-lactams, aminoglycosides, and quinolones. *Salmonella* resistance to ampicillin and cephalosporins is often due to β-lactamase production. Ciprofloxacin resistance results from mutations in the *gyrA* and *parC* genes, while plasmid-mediated quinolone resistance and efflux pumps contribute to low-level quinolone resistance. AMR varies by region: China reports ampicillin resistance at 73.4%, third-generation cephalosporins at 20%, fluoroquinolones at 16.2%, and MDR at 40–81%. Europe shows ampicillin resistance at 25.2%, third-generation cephalosporins at 1.1%, fluoroquinolones at 14.9%, and MDR at 22.6%. The USA has ampicillin resistance at 6.6%, third-generation cephalosporins at 3%, fluoroquinolones at 3%, and MDR at 10.3% [102]. The prevalence of MDR isolates in sub-Saharan Africa reached 75% [103]. Finally, fluoroquinolone-resistant NTS indirectly increases resistance in typhoidal *Salmonella* by contributing to the prevalence of resistance genes. Shared genes increase the risk of resistance transfer, compromising the effectiveness of fluoroquinolone in treating typhoid fever. This is why the WHO added fluoroquinolone-resistant NTS to the high-priority category of bacterial pathogens of public health importance to guide research, development, and strategies to prevent and control antimicrobial resistance [104].

##### *Campylobacter* spp.

In 2023, campylobacteriosis remained the most commonly reported zoonosis in the EU, accounting for 58.9% of confirmed human cases [99], with a notification rate of 45.7 cases per 100,000 population. The number of confirmed cases is shown in Table 2. Campylobacteriosis in humans is primarily due to the consumption of undercooked poultry, whereas outbreaks are mostly associated with the consumption of raw milk or dairy products. *Campylobacter* is also present in the intestines of companion animals (such as dogs and cats), and interaction with puppies infected with *Campylobacter* has been linked to transmission.

*Campylobacter* is exposed to antibiotics used in food-producing animals, companion animals, and humans, which has led to the development of various resistance mechanisms. As a result, antibiotic-resistant *Campylobacter* is becoming more common, threatening the effectiveness of antibiotic treatments and posing a significant public health risk. Macrolides are the preferred antibiotics for treating campylobacteriosis. However, increasing rates of macrolide resistance, particularly in *C. coli* strains from China, Spain, and Peru, have raised concerns about relying on macrolides as the primary treatment in these regions [105]. Fluoroquinolones and tetracycline are alternative options but are not recommended for children. Over the past decade, resistance to ciprofloxacin and tetracycline has quickly risen in *Campylobacter* spp. among humans in Europe, while resistance to erythromycin has remained relatively low. The use of fluoroquinolone antibiotics has contributed to the emergence of resistance, with reports indicating a 75–90% prevalence of fluoroquinolone resistance in clinical *Campylobacter* strains across various countries [106,107]. Aminoglycosides like gentamicin are effective for systemic infections, as *Campylobacter* is generally susceptible to them. However, the emergence of novel aminoglycoside resistance genes and multidrug resistance islands threatens their clinical effectiveness [108]. Due to increasing rates of MDR *Campylobacter* spp., there are suggestions to include amoxicillin–clavulanic acid or fosfomycin tromethamine as treatment options. Future studies will evaluate their effectiveness.

##### Diarrheagenic *E. coli*

*Escherichia coli* is the predominant commensal gut bacterium. However, there are strains capable of causing diseases in the human intestinal tract and are designated as diarrheagenic *E. coli* (DEC), which is subclassified into seven different pathotypes: Shiga toxin-producing *E. coli* (STEC), enteropathogenic *E. coli* (EPEC), enterotoxigenic *E. coli* (ETEC), enteroaggregative *E. coli* (EAEC), enteroinvasive *E. coli* (EIEC), diffusely adherent *E. coli* (DAEC), and the recently identified, though uncommon, adherent-invasive *E. coli* (AIEC) [109].

The risk factors associated with gut colonization and the spread of infection caused by diarrheagenic *E. coli* (DEC) in humans include poor sanitation and hygiene practices such as open defecation, lack of clean water, inadequate hand washing facilities, malnutrition, HIV/AIDS infections, overcrowded living conditions such as in refugee camps and slums, contact with infected individuals and animals, and contaminated food and water [110,111].

If indicated, antibiotic treatment includes fluoroquinolone, azithromycin, or rifaximin. In addition, trimethoprim–sulfamethoxazole and third-generation cephalosporins are also in use. Although studies investigating the antimicrobial resistance of DEC in Europe are scarce, studies on DEC in Asia have shown that the highest resistance of DEC was to penicillin-based antibiotics, with amoxicillin exhibiting 80.9% resistance, followed by ampicillin at 73.5% [112]. In South Africa, the antimicrobial resistance of DEC was as follows: amikacin (95%), gentamycin (93%), meropenem (91%), chloramphenicol (90%), norfloxacin (88%), nitrofurantoin (87%), imipenem (84%), and polymyxin B (83%), with ceftazidime, cefotaxime, cephalothin, and nalidixic acids demonstrating susceptibilities slightly above average (62, 55, 62, and 58%, respectively) [113]. A growing number of ARGs have been found in *E. coli*, many of which are situated in mobile genetic elements, allowing them to be transmitted between animals, humans, and the environment.

*Bla_TEM_* was the most commonly detected resistance determinant among the DEC isolates in South Africa, with an average of 33% of the isolates carrying this resistance factor [113], supporting the findings of the study by Zhou et al. in central China [114].

### 4.2. Non-Pathogenic and Opportunistic Bacteria as the Source of Antimicrobial Resistance in Food

Besides pathogenic bacteria, non-pathogenic and opportunistic bacteria should be included in the list of public health concerns, as they serve as reservoirs for AMR genes, which can be transferred to pathogenic bacteria directly of via the members of the gut microbiome. Gene transfer occurs through three main mechanisms. Bacteria can directly take up DNA from the environment or with the assistance of vectors like conjugative plasmids, conjugative integrative elements, or bacteriophages [115]. Examples of these bacterial species carrying antibiotic resistance genes are given in Table 3.

## 5. Conclusions

There is still a high presence of AMR, with possible transmission-mediated routes between different ecological niches. While the trends in the prevalence of foodborne pathogens have changed over time, the burden of the ecological niches with resistance genes, primarily in commensal microorganisms, is of concern. The implementation of the harmonized surveillance of AMU and AMR would provide comprehensive insights into the actual status of resistance and further interventions leading to its reduction. WGS should be used as a genome-based tool necessary for tracking AMR within the One Health concept. The development of publicly available repositories for AMR minimum metadata from human and non-human samples, not only for pathogenic microorganisms but also for non-pathogenic ones, will enable more comprehensive AMR surveillance, monitoring of transmission-mediated pathways, and identification of AMR sources to be carried out, potentially resulting in its elimination.

## Figures and Tables

**Table 1:** Antimicrobial sales for food-producing animals in the EU and USA in 2023 [79,80].

Antimicrobial Classes	EU		USA	
	Antimicrobial Sales (tonnes)	%	Antimicrobial Sales (tonnes)	%
Penicillins	1354.16	31.4	614.62	10.2
Tetracyclines	931.52	21.6	4051.12	66.9
Sulfonamides	435.57	10.1	263.09	4.3
Macrolides	414.01	9.6	530.59	8.8
Aminoglycosides	297.57	6.9	320.63	5.3
Lincosamides	228.57	5.3	149.75	2.5
Other classes *	219.94	5.1	95.29	1.6
Fluoroquinolones	99.19	2.3	26.91	0.4
Pleuromutilins	146.63	3.4	-	-
Polymixins	116.44	2.7	-	-
Trimethoprim	69.00	1.6	-	-
Total	4312.60	100.0	6051.99	100.0

* Other classes: amphenicols, cephalosporins, other quinolones, and other antibacterials.

**Table 2:** The most common zoonotic foodborne pathogens—EU, 2023.

Bacteria	Confirmed Human Cases (N)	Hospitalizations and Proportion of Hospitalized Cases (N)	Deaths (N)
*Campylobacter* spp.	148,181	90	0
*Salmonella* spp.	77,486	1726	16
*STEC infections*	10,217	48	1
*Yersinia* spp.	8738	9	0
*Listeria monocytogenes*	2952	84	11
*Brucella* spp.	259	0	0
*Mycobacterium bovis* and *M. caprae (Mycobacterium causing zoonotic tuberculosis)*	138	0	0

**Table 3:** Non-pathogenic and opportunistic bacteria in fresh, ready-to-eat, and fermented food as antimicrobial resistance gene reservoirs.

Bacterial Origin	Bacteria	Antibiotic Class and Corresponding Resistance Genes	Reference
	Gram-positive		
Street kebab, raw buffalo meat	*Staphylococcus* spp.	β-lactam → *mecA* Fluoroquinolone → *qnrB/S* Sulfonamide → *sul1*	[116]
Fish, crustacean	*Enterococcus* spp.	Aminoglycoside → *aac(6′)-Ie-aph(2″)-Ia* β-lactam → *blaZ* Fluoroquinolone → *gyrA* Glycopeptide → *vanA*, *vanB* Macrolide → *ermB* Oxazolidinone → *optrA* Tetracycline → *tetM/K*	[117]
Yogurt	*Streptococcus thermophiles*, *Lactobacillus bulgaricus*	Aminoglycoside → *AacA-D*, *strA*, *strB* β-lactam → *blaZ* Fluoroquinolone → *gyrA* Lincosamide → *linA*, *cat1* Macrolide → *ermA* Nitrofuran → *nfsA* Rifamycin → *rpoB* Tetracycline → *tetM/K*	[118]
Nono	*Enterococcus thailandicus*, *Streptococcus infantarius*	Aminoglycoside → *aadE* Tetracycline → *tetS/M*	[119]
Ready-to-eat food	*Enterococcus* spp. (*E. faecium*, *E. faecalis)*	Aminoglycoside → *aac(6′)-Ie-aph(2′′)-Ia*, *ant(6′)-Ia* Macrolide → *mefA/E*, *ermB* Tetracycline → *tetK/L/M*	[120]
Pork and llama sausages	*Latilactobacillus sakei*, *Lactiplantibacillus plantarum*, *Staphylococcus* spp. (*S. xylosus*, *S. equorum*, *S. saprophyticus*)	Macrolide → *ermA/B/C* Tetracycline → *tetK/L/M/S/W*	[121]
	Gram-negative		
Tomatoes, carrots, iceberg lettuce, strawberries, coriander	*Enterobacter* spp. (*E. cloacae*, *E. ludwigii*, *E. asburiae*, *E. kobei)*, *Lelliottia amnigena*, *Morganella morganii*, *Serratia* spp. (*S. fonticola*, *S. odorifera*), *Pseudomonas aeruginosa*	Aminoglycoside → *aadE* β-lactam → *bla_TEM_/_CTX-M_/_SHV_/_CMY_/_OXA-48_* Fluoroquinolone → *qnrS* Sulfonamide → *sul1* Tetracycline → *tetW*	[122]
Minced meat, diced meat, chicken meat, mutton meat	*Klebsiella pneumoniae*	Aminoglycoside → *aph(3′)-VI*, *aph(3′)Ia*, *aph(3′′)-Ib*, *aph(6)-Id*, *aac (3)-IIa*, *aac (6′)-Ib*, *aadA1*, *aac(6′)-Ib-c*, *rmtF* Amphenicol → *catB3* β-lactam → *bla_NDM-1_/_NDM-5/OXA-48/OXA-9_/_OXA-1_/_OXA-10_/_CTX-M-15_/_SHV_-_67_/_SHV-1_/_SHV-148_/_CTX-M-14b_/_TEM-1A_/_TEM-1C_/_CTX-M-82_/_CTX-M-194_*/*_TEM_*_-*1B*_ Fluoroquinolone → *qnrB1*, *OqxA*, *OqxB* Fosfomycin → *fosA*, *fosA6* Sulfonamide → *sul1/2* Tetracycline → *tetA* Trimethoprim → *dfrA5*	[123]
Fresh mussels and oysters	*Aeromonas* spp.	Aminoglycoside → *aadA2*, *dfrA12*, *aph(3″)-Ib*, *aph(6)-Id* Amphenicol → *catA3*, *catIII* β-lactam → *ampS*, *ampH*, *bla_FOX_/_CEPH-A3_/_MOX_/oxa/*_AQU_, *cphA3/4/6/7*, *imiH*Polymyhin → *mcr3* Sulfonamide → *sul1/2* Tetracycline → *tetA/E/Y* Trimethoprim → *dfrA15*	[124]
Fresh cabbage, spinach, lettuce	*Klebsiella pneumoniae*, *Escherichia coli*, *Pseudomonas aeruginosa*	β-lactam → *bla_KPC-2_/_NDM-1_/_VIM-2_*	[125]
Fresh vegetables	*Pseudomonas putida*	β-lactam → *BepC*, *bcr*, *Mdtl*, *AcrAB-TolC*, *MexEF-OprN* Quinolone → *Mdtl*, *AcrAB-TolC*, *MexEF-OprN* Tetracycline → *Mdtl*, *AcrAB-TolC*, *MexEF-OprN*	[126]

## References

[1] Martak D., Henriot C.P., Hocquet D. (2024). Environment, animals, and food as reservoirs of antibiotic-resistant bacteria for humans: One health or more?. Infect. Dis. Now.

[2] Founou L.L., Founou R.C., Essack S.Y. (2021). Antimicrobial resistance in the farm-to-plate continuum: More than a food safety issue. Future Sci. OA.

[3] Sweileh W.M. (2021). Global research activity on antimicrobial resistance in food-producing animals. Arch. Public Health.

[4] Pokharel S., Shrestha P., Adhikari B. (2020). Antimicrobial use in food animals and human health: Time to implement ‘One Health’ approach. Antimicrob. Resist. Infect. Control.

[5] Hoffmann S., White A.E., McQueen R.B., Ahn J.W., Gunn-Sandell L.B., Scallan Walter E.J. (2024). Economic Burden of Foodborne Illnesses Acquired in the United States. Foodborne Pathog. Dis..

[6] Fischer M.M., Bild M. (2019). Hospital use of antibiotics as the main driver of infections with antibiotic-resistant bacteria–a reanalysis of recent data from the European Union. bioRxiv.

[7] Chang Q., Wang W., Regev-Yochay G., Lipsitch M., Hanage W.P. (2015). Antibiotics in agriculture and the risk to human health: How worried should we be?. Evol. Appl..

[8] Magouras I., Carmo L.P., Stärk K.D., Schüpbach-Regula G. (2017). Antimicrobial usage and-resistance in livestock: Where should we focus?. Front. Vet. Sci..

[9] Bennani H., Mateus A., Mays N., Eastmure E., Stärk K.D., Häsler B. (2020). Overview of evidence of antimicrobial use and antimicrobial resistance in the food chain. Antibiotics.

[10] Hudson J.A., Frewer L.J., Jones G., Brereton P.A., Whittingham M.J., Stewart G. (2017). The agri-food chain and antimicrobial resistance: A review. Trends Food Sci. Technol..

[11] Lechner I., Freivogel C., Stärk K.D., Visschers V.H. (2020). Exposure pathways to antimicrobial resistance at the human-animal interface—A qualitative comparison of Swiss expert and consumer opinions. Front. Public Health.

[12] Laxminarayan R., Duse A., Wattal C., Zaidi A.K., Wertheim H.F., Sumpradit N., Vlieghe E., Hara G.L., Gould I.M., Goossens H. (2013). Antibiotic resistance—The need for global solutions. Lancet Infect. Dis..

[13] Torres R.T., Carvalho J., Fernandes J., Palmeira J.D., Cunha M.V., Fonseca C. (2021). Mapping the scientific knowledge of antimicrobial resistance in food-producing animals. One Health.

[14] Verhaegen M., Bergot T., Liebana E., Stancanelli G., Streissl F., Mingeot-Leclercq M.P., Mahillon J., Bragard C. (2023). On the use of antibiotics to control plant pathogenic bacteria: A genetic and genomic perspective. Front. Microbiol..

[15] Auerbach E.A., Seyfried E.E., McMahon K.D. (2007). Tetracycline resistance genes in activated sludge wastewater treatment plants. Water Res..

[16] Zlatković N., Gašić K., Kuzmanović N., Prokić A., Ivanović M., Ţivković S., Obradović A. (2022). Polyphasic Characterization of *Acidovorax citrulli* Strains Originating from Serbia. Agronomy.

[17] Stockwell V.O., Duffy B. (2012). Use of antibiotics in plant agriculture. Rev. Sci. Tech..

[18] Batuman O., Britt-Ugartemendia K., Kunwar S., Yilmaz S., Fessler L., Redondo A., Chumachenko K., Chakravarty S., Wade T. (2024). The Use and Impact of Antibiotics in Plant Agriculture: A Review. Phytopathology.

[19] Sundin G.W., Wang N. (2018). Antibiotic Resistance in Plant-Pathogenic Bacteria. Annu. Rev. Phytopathol..

[20] McManus P.S., Stockwell V.O., Sundin G.W., Jones A.L. (2002). Antibiotic use in plant agriculture. Annu. Rev. Phytopathol..

[21] Vidaver A.K. (2002). Uses of antibiotics in plant agriculture. Clin. Infect. Dis..

[22] McManus P., Jones A.L. (1994). Epidemiology and Genetic Analysis of Streptomycin-Resistant *Erwinia amylovora* from Michigan and Evaluation of Oxytetracycline for Control. Phytopathology.

[23] Halbert S. The discovery of huanglongbing in Florida. Proceedings of the 2nd International Citrus Canker and Huanglongbing Research Workshop.

[24] da Graça J.V., Kunta M., Sétamou M., Rascoe J., Li W., Nakhla M.K., Salas B., Bartels D.W. (2015). Huanglongbing in Texas: Report on the first detections in commercial citrus. J. Citr. Pathol..

[25] Vincent C.I., Hijaz F., Pierre M., Killiny N. (2022). Systemic uptake ofoxytetracycline and streptomycin in huanglongbing-affected citrus groves after foliar application and trunk injection. Antibiotics.

[26] Wise J.C., VanWoerkom A.H., Aćimović S.G., Sundin G.W., Cregg B.M., Vandervoort C. (2014). Trunk Injection: A Discriminating Delivering System for Horticulture Crop IPM. Entomol. Ornithol. Herpetol..

[27] Chopra I., Roberts M. (2001). Tetracycline antibiotics: Mode of action, applications, molecular biology, and epidemiology of bacterial resistance. Microbiol. Mol. Biol. Rev..

[28] U.S. EPA. Kasugamycin. 20CA01 (2020). Human Health Risk Assessment for the Proposed Section 18 Specific Exemption for Use on Almonds.

[29] Shtienberg D., Zilberstaine M., Oppenheim D., Herzog Z., Manulis S., Shwartz H., Kritzman K. (2001). Efficacy of oxolinic acid and other bactericides in suppression of *Erwinia amylovora* in pear orchards in Israel. Phytoparasitica.

[30] Nandakumar R., Shahjahan A.K.M., Yuan X.L., Dickstein E.R., Groth D.E., Clark C.A., Cartwright R.D., Rush M.C. (2009). *Burkholderia glumae* and *B. gladioli* Cause Bacterial Panicle Blight in Rice in the Southern United States. Plant Dis..

[31] Kleitman F., Shtienberg D., Blachinsky D., Oppenheim D., Zilberstaine M., Dror O., Manulis S. (2005). *Erwinia amylovora* populations resistant to oxolinic acid in Israel: Prevalence, persistence and fitness. Plant Pathol..

[32] McManus P., Stockwell V. (2000). Antibiotics for Plant Diseases Control: Silver Bullets or Rusty Sabers?. APS Net Features..

[33] Šević M., Gašić K., Ignjatov M., Mijatović M., Prokić A., Obradović A. (2019). Integration of biological and conventional treatments in control of pepper bacterial spot. Crop. Prot..

[34] Bonetta S., Di Cesare A., Pignata C., Sabatino R., Macri M., Corno G., Panizzolo M., Bonetta S., Carraro E. (2023). Occurrence of antibiotic-resistant bacteria and resistance genes in the urban water cycle. Environ. Sci. Pollut. Res..

[35] Kittinger C., Lipp M., Folli B., Kirschner A., Baumert R., Galler H., Grisold A.J., Luxner J., Weissenbacher M., Farlneitner A.H. (2016). *Enterobacteriaceae* Isolated from the River Danube: Antibiotic Resistances, with a Focus on the Presence of ESBL and Carbapenemases. PLoS ONE.

[36] Lepuschitz S., Schill S., Stoager A., Pekard-Amenitsch S., Huhulescu S., Inreiter N., Hartl R., Kerschner H., Sorcharg S., Springer B. (2019). Whole genome sequencing reveals resemblance between ESBL-producing and carbapenem resistant *Klebsiella pneumonia* isolates from Austrian rivers and clinical isolates from hospitals. Sci. Total Environ..

[37] Hu Y., Jin L., Zhao Y., Jiang L., Yao S., Zhou W., Kuangfei L., Changzheng C. (2021). Annual trends and health risks of antibiotics and antibiotic resistance genes in a drinking water source in East China. Sci. Total Environ..

[38] Yang F.X., Mao D.Q., Zhou H., Luo Y. (2016). Prevalence and fate of carbapenemase genes in a wastewater treatment plant in Northern China. PLoS ONE.

[39] Gundogdu A., Jenisson A.V., Smith H.V., Stratton H., Katouli M. (2013). Extended-spectrum β-lactamase producing Escherichia coli in hospital wastewaters and sewage treatment plants in Queensland, Australia. Can. J. Microbiol..

[40] Bergeron S., Boopathy R., Nathaniel R., Corbin A., Lafleur G. (2015). Presence of antibiotic resistant bacteria and antibiotic resistance genes in raw source water and treated drinking water. Int. Biodeterior. Biodegrad..

[41] Rizzo L., Manaia C., Merlin C., Schwartz T., Dagot C., Ploy M.C., Michael I., Fatta-Kassino D. (2013). Urban wastewater treatment plants as hotspots for antibiotic resistant bacteria and genes spread into the environment: A review. Sci. Total Environ..

[42] Ghernaout D., Elboughdiri N. (2020). Antibiotic Resistance in Water Medium: Background, Facts, and Trends. Appl. Eng..

[43] Sugden R., Kelly R., Davies S. (2016). Combating antimicrobial resistance globally. Nat. Microbiol..

[44] Van Boeckel T.P., Gandra S., Ashok A. (2014). Global antibiotic consumption 2000 to 2010: An analysis of national pharmaceutical sales data. Lancet Infect. Dis..

[45] Casanova L.M., Hill V.R., Sobsey M.D. (2019). Antibiotic-resistance *Salmonella* in swine wastes and farm surface water. Lett. Appl. Microbiol..

[46] Federigi I., Bonetta S., Tesauro M., De Giglio O., Conti G.O., Atomsa N.T., Bagordo F., Bonetta S., Consonni M., Diella G. (2024). A systematic scoping review of antibiotic-resistance in drinking tap water. Environ. Res..

[47] Mompelat S., Thomas O., Le Bot B. (2011). Contamination Levels of Human Pharmaceutical Compounds in French Surface and Drinking Water. J. Environ. Monit..

[48] Vulliet E., Cren-Olive C., Grenier-Loustalot M.-F. (2011). Occurrence of Pharmaceuticals and Hormones in Drinking Water Treated from Surface Waters. Environ. Chem. Lett..

[49] Mi R., Patidar R., Farenhorst A., Cai Z., Sepehri S., Khafipour E., Kumar A. (2019). Detection of fecal bacteria and antibiotic resistance genes in drinking water collected from three First Nations communities in Manitoba, Canada. FEMS Microbiol. Lett..

[50] Neudorf K.D., Huang Y.N., Ragush C.M., Yost C.K., Jamieson R.C., Hansen L.T. (2017). Antibiotic resistance genes in municipal wastewater treatment systems and receiving waters in Arctic Canada. Sci. Total Environ..

[51] Guo X., Xiaojun L., Zhang A., Yan Z., Chen S., Wang N. (2019). Antibiotic contamination in a typical water-rich city in southeast China: A concern for drinking water resource safety. J. Environ. Sci. Health Part B.

[52] Bergeron S., Boopathy R., Rujkumar N., Corbin A., Lafleur G. (2017). Presence of antibiotic resistance genes in raw source water of a drinking water treatment plant in a rural community of USA. Int. Biodeterior. Biodegrad..

[53] Bird K., Boopathy R., Nathaniel R., LaFleur G. (2019). Water pollution and observation of acquired antibiotic resistance in Bayou Lafourche, a major drinking water source in Southeast Louisiana, USA. Environ. Sci. Pollut. Res..

[54] Rodriguez-Mozaz S., Chamorro S., Marti E., Huerta B., Gros M., Sanchez-Melsio A., Borrego C.M., Barcelo D., Balcazar J.L. (2015). Occurrence of antibiotics and antibiotic resistance genes in hospital and urban wastewaters and their impact on the receiving river. Water Res..

[55] Dong X., Sun S., Xu L. (2020). Research progress of antibiotic resistance Genes (ARGs) pollution in drinking water source. IOP Conf. Ser. Earth Environ. Sci..

[56] Hubbard L.E., Kolpin D.W., Givens C.E., Blackwell B.R., Bradley P., Gray J.L., Lane R.F., Masoner J.R., McCleskey R.B., Romanok K.M. (2022). Food, Beverage, and Feedstock Processing Facility Wastewater: A Unique and Underappreciated Source of Contaminants to U.S. Streams. Environ. Sci. Technol..

[57] Taylor N.G., Verner-Jeffreys D.W., Baker-Austin C. (2011). Aquatic systems: Maintaining, mixing and mobilizing antimicrobial resistance?. Trends Ecol. Evol..

[58] Carattoli A. (2008). Animal reservoirs for extended spectrum beta-lactamase producers. Clin. Microbiol. Infect..

[59] Khan F.A., Hellmark B., Ehricht R., Söderquist B., Jass J. (2018). Related carbapenemase producing *Klebsiella* isolates detected in both a hospital and associated aquatic environment in Sweden. Eur. J. Clin. Microbiol. Infect. Dis..

[60] Sun Y., Guo Y., Shi M., Qiu T., Gao M., Tian S., Wang X. (2020). Effect of antibiotic type and vegetable species on antibiotic accumulation in soil-vegetable system, soil microbiota, and resistance genes. Chemosphere.

[61] Yao D., Chang Y., Wang W., Sun L., Liu J., Zhao H., Zhang W. (2022). The Safety of Consuming Water Dropwort Used to Purify Livestock Wastewater Considering Accumulated Antibiotics and Antibiotic Resistance Genes. Antibiotics.

[62] Mohamad Z.A., Bakon S.K., Jamila М.А.Ј., Daud N., Ciric L., Ahmad N., Muhamad N.A. (2022). Prevalence of Antibiotic-Resistant Bacteria and Antibiotic-Resistant Genes and the Quantification of Antibiotics in Drinking Water Treatment Plants of Malaysia: Protocol for a Cross-sectional Study. JMIR Res. Protoc..

[63] Reuther R., Inyang H.I., Daniels J.L. (2009). Lake and river sediment monitoring. Environmental monitoring. Encyclopedia of Life Support Systems.

[64] Filipic B., Novovic K., Studholme D.J., Malesevic M., Mirkovic N., Kojic M., Jovcic B. (2020). Shotgun metagenomics reveals differences in antibiotic resistance genes among bacterial communities in Western Balkans glacial lakes sediments. J. Water Health.

[65] Almansour A.M., Alhadlaq M.A., Alzahrani K.O., Mukhtar L.E., Alharbi A.L., Alajel S.M. (2023). The silent threat: Antimicrobial-resistant pathogens in food-producing animals and their impact on public health. Microorganisms.

[66] Van Boeckel T.P., Glennon E.E., Chen D., Gilbert M., Robinson T.P., Grenfell B.T., Levin S.A., Bonhoeffer S., Laxminarayan R. (2017). Reducing antimicrobial use in food animals. Science.

[67] Roca I., Akova M., Baquero F., Carlet J., Cavaleri M., Coenen S., Cohen J., Findlay D., Gyssens I., Heure O.E. (2015). The global threat of antimicrobial resistance: Science for intervention. New Microbes New Infect..

[68] Aminov R.I. (2009). The role of antibiotics and antibiotic resistance in nature. Environ. Microbiol..

[69] Van Boeckel T.P., Brower C., Gilbert M., Grenfell B.T., Levin S.A., Robinson T.P., Teillant A., Laxminarayan R. (2015). Global trends in antimicrobial use in food animals. Proc. Natl. Acad. Sci. USA.

[70] Tiseo K., Huber L., Gilbert M., Robinson T.P., Van Boeckel T.P. (2020). Global trends in antimicrobial use in food animals from 2017 to 2030. Antibiotics.

[71] O’Neill J. Tackling Drug-Resistant Infections Globally: Final Report and Recommendations. https://amr-review.org/sites/default/files/160518_Final%20paper_with%20cover.pdf.

[72] Vikesland P., Garner E., Gupta S., Kang S., Maile-Moskowitz A., Zhu N.I. (2019). Differential drivers of antimicrobial resistance across the world. Acc. Chem. Res..

[73] Looft T., Johnson T.A., Allen H.K., Bayles D.O., Alt D.P., Stedtfeld R.D., Sul W.J., Stedtfeld T.M., Chai B., Cole J.R. (2012). In-feed antibiotic effects on the swine intestinal microbiome. Proc. Natl. Acad. Sci. USA.

[74] Rhouma M., Beaudry F., Thériault W., Letellier A. (2016). Colistin in pig production: Chemistry, mechanism of antibacterial action, microbial resistance emergence, and one health perspectives. Front. Microbiol..

[75] Rhouma M., Soufi L., Cenatus S., Archambault M., Butaye P. (2022). Current insights regarding the role of farm animals in the spread of antimicrobial resistance from a one health perspective. Vet. Sci..

[76] Aidara-Kane A., Angulo F.J., Conly J.M., Minato Y., Silbergeld E.K., McEwen S.A., Collignon P.J. (2018). WHO Guideline Development Group Hanan Balkhy Peter Collignon John Conly Cindy Friedman Aidan Hollis Samuel Kariuki Hyo-Sun Kwak Scott McEwen Gérard Moulin Antoinette Ngandjio Bernard Rollin Flávia Rossi David Wallinga. World Health Organization (WHO) guidelines on use of medically important antimicrobials in food-producing animals. Antimicrob. Resist. Infect. Control.

[77] McEwen S.A., Collignon P.J. (2018). Antimicrobial resistance: A one health perspective. Antimicrobial Resistance in Bacteria from Livestock and Companion Animals.

[78] Roy J.P., Archambault M., Desrochers A., Dubuc J., Dufour S., Francoz D., Paradis M.È., Rousseau M. (2020). New Quebec regulation on the use of antimicrobials of very high importance in food animals: Implementation and impacts in dairy cattle practice. Can. Vet. J..

[79] European Sales and Use of Antimicrobials for Veterinary Medicine (ESUAvet) Annual Surveillance Report for 2023, (EMA/CVMP/ESUAVET/80289/2025). https://www.ema.europa.eu/en/documents/report/european-sales-use-antimicrobials-veterinary-medicine-annual-surveillance-report-2023_en.pdf.

[80] US Food and Drug Administration (2023). 2023 Summary Report on Antimicrobials Sold or Distributed for Use in Food-Producing Animals.

[81] Werner N., McEwen. S., Kreienbrock L. (2018). Monitoring antimicrobial drug usage in animals: Methods and applications. Antimicrobial Resistance in Bacteria from Livestock and Companion. Animals.

[82] Tang K.L., Caffrey N.P., Nóbrega D.B., Cork S.C., Ronksley P.E., Barkema H.W., Polachek A.J., Ganshorn H., Sharma N., Kellner J.D. (2017). Restricting the use of antibiotics in food-producing animals and its associations with antibiotic resistance in food-producing animals and human beings: A systematic review and meta-analysis. Lancet Planet. Health.

[83] Khater D.F., Lela R.A., El-Diasty M., Moustafa S.A., Wareth G. (2021). Detection of harmful foodborne pathogens in food samples at the points of sale by MALDT-TOF MS in Egypt. BMC Res. Notes.

[84] Aerts M., Battisti A., Hendriksen R., Kempf I., Teale C., EFSA (2019). Technical specifications on harmonised monitoring of antimicrobial resistance in zoonotic and indicator bacteria from food-producing animals and food. EFSA J..

[85] Mthembu T.P., Zishiri O.T., El Zowalaty M.E. (2021). Genomic characterization of antimicrobial resistance in food chain and livestock-associated Salmonella species. Animals.

[86] Chika E. (2018). A three-year study on the prevalence and antibiotic susceptibility pattern of Escherichia coli isolated from cloacal swabs of wild and domestic birds in Ebonyi State, Nigeria. EC Microbiol..

[87] Fetahagić M., Ibrahimagić A., Uzunović S., Beader N., Elveđi-Gašparović V., Luxner J., Gladan M., Bedenić B. (2021). Detection and characterisation of extended-spectrum and plasmid-mediated AmpC β-lactamase produced by Escherichia coli isolates found at poultry farms in Bosnia and Herzegovina. Arh. Hig. Rada Toksikol..

[88] Kowalska-Krochmal B., Dudek-Wicher R. (2021). The minimum inhibitory concentration of antibiotics: Methods, interpretation, clinical relevance. Pathogens.

[89] Mellmann A., Bletz S., Böking T., Kipp F., Becker K., Schultes A., Prior K., Harmsen D. (2016). Real-time genome sequencing of resistant bacteria provides precision infection control in an institutional setting. J. Clin. Microbiol..

[90] Kozyreva V.K., Crandall J., Sabol A., Poe A., Zhang P., Concepción-Acevedo J., Schroeder M.N., Wagner D., Higa J., Trees E. (2016). Laboratory investigation of Salmonella enterica serovar Poona outbreak in California: Comparison of pulsed-field gel electrophoresis (PFGE) and whole genome sequencing (WGS) results. PLoS Curr..

[91] Whole Genome Sequencing as a Tool to Strengthen Foodborne Disease Surveillance and Response (2023). Module 2. Whole Genome Sequencing in Foodborne Disease Outbreak Investigations.

[92] Wedley A.L., Dawson S., Maddox T.W., Coyne K.P., Pinchbeck G.L., Clegg P., Nuttall T., Kirchner M., Williams N.J. (2017). Carriage of antimicrobial resistant Escherichia coli in dogs: Prevalence, associated risk factors and molecular characteristics. Vet. Microbiol..

[93] Abukhattab S., Hosch S., Abu-Rmeileh N.M.E., Hasan S., Vonaesch P., Crump L., Hattendorf J., Daubenberger C., Zinsstag J., Schindler T. (2023). Whole-genome sequencing for One Health surveillance of antimicrobial resistance in conflict zones: A case study of *Salmonella* spp. and *Campylobacter* spp. in the West Bank, Palestine. Appl. Environ. Microbiol..

[94] Birgy A., Madhi F., Jung C., Levy C., Cointe A., Bidet P., Hobson C.A., Bechet S., Sobral E., Vuthien H. (2020). Diversity and trends in population structure of ESBL-producing Enterobacteriaceae in febrile urinary tract infections in children in France from 2014 to 2017. J. Antimicrob. Chemother..

[95] World Health Organisation Foodborne Diseases Estimates. https://www.who.int/data/gho/data/themes/who-estimates-of-the-global-burden-of-foodborne-diseases.

[96] World Health Organization (2017). Diarrhoeal Disease.

[97] European Food Safety Authority (EFSA), European Centre for Disease Prevention and Control (ECDC) (2023). The European Union One Health 2022 Zoonoses Report. EFSA J..

[98] World Health Organization Salmonella (Non-Typhoidal), Key Facts. https://www.who.int/news-room/fact-sheets/detail/salmonella-(non-typhoidal).

[99] EFSA and ECDC (European Food Safety Authority and European Centre for Disease Prevention and Control) (2024). The European Union One Health 2023 Zoonoses report. EFSA J..

[100] Interagency Food Safety Analytics Collaboration (2021). Foodborne Illness Source Attribution Estimates for 2019 for Salmonella, Escherichia coli O157, Listeria Monocytogenes and Campylobacter Using Multi-Year Outbreak Surveillance Data, United States.

[101] Marchello C.S., Birkhold M., Crump J.A. (2022). Vacc-iNTS consortium collaborators. Complications and mortality of non-typhoidal salmonella invasive disease: A global systematic review and meta-analysis. Lancet Infect. Dis..

[102] Sima C.M., Buzilă E.R., Trofin F., Păduraru D., Luncă C., Duhaniuc A., Dorneanu O.S., Nastase E.V. (2024). Emerging Strategies against Non-Typhoidal *Salmonella*: From Pathogenesis to Treatment. Curr. Issues Mol. Biol..

[103] Tack B., Vanaenrode J., Verbakel J.Y., Toelen J., Jacobs J. (2020). Invasive non-typhoidal *Salmonella* infections in sub-Saharan Africa: A systematic review on antimicrobial resistance and treatment. BMC Med..

[104] (2024). WHO Bacterial Priority Pathogens List, 2024: Bacterial Pathogens of Public Health Importance to Guide Research, Development and Strategies to Prevent and Control Antimicrobial Resistance.

[105] Du Y., Wang C., Ye Y., Liu Y., Wang A., Li Y., Zhou X., Pan H., Zhang J., Xu X. (2018). Molecular Identification of Multidrug-Resistant *Campylobacter* Species From Diarrheal Patients and Poultry Meat in Shanghai, China. Front. Microbiol..

[106] Schiaffino F., Colston J.M., Paredes-Olortegui M., François R., Pisanic N., Burga R., Peñataro-Yori P., Kosek M.N. (2019). Antibiotic Resistance of *Campylobacter* Species in a Pediatric Cohort Study. Antimicrob. Agents Chemother..

[107] Signorini M.L., Rossler E., Díaz David D.C., Olivero C.R., Romero-Scharpen A., Soto L.P., Astesana D.M., Berisvil A.P., Zimmermann J.A., Fusari M.L. (2018). Antimicrobial Resistance of Thermotolerant Campylobacter Species Isolated from Humans, Food-Producing Animals, and Products of Animal Origin: A Worldwide Meta-Analysis. Microb. Drug Resist..

[108] Zhao S., Mukherjee S., Chen Y., Li C., Young S., Warren M., Abbott J., Friedman S., Kabera C., Karlsson M. (2015). Novel gentamicin resistance genes in Campylobacter isolated from humans and retail meats in the USA. J. Antimicrob. Chemother..

[109] Gomes T.A., Elias W.P., Scaletsky I.C., Guth B.E., Rodrigues J.F., Piazza R.M., Ferreira L.C., Martinez M.B. (2016). Diarrheagenic Escherichia coli. Braz. J. Microbiol..

[110] Gautam O., Curtis V. (2021). Food hygiene practices of rural women and microbial risk for children: Formative research in Nepal. Am. J. Trop. Med. Hyg..

[111] Hoffmann V., Simiyu S., Sewell D., Tsai K., Cumming O., Mumma J., Baker K.K. (2022). Milk product safety and household food hygiene influence bacterial contamination of infant food in Peri-Urban Kenya. Front. Public Health.

[112] Salleh M.Z., Mik Zuraina N.M., Hajissa K., Ilias M.I., Deris Z.Z. (2022). Prevalence of Multidrag Resistant Diarrheagenic Escherichia coli in Asia: A Systematic Review and Meta Analysis. Antibiotics.

[113] Msolo L., Iweriebor B.C., Okoh A.I. (2020). Antimicrobial Resistance Profiles of Diarrheagenic *E. coli* (DEC) and *Salmonella* Species Recovered from Diarrheal Patients in Selected Rural Communities of the Amathole District Municipality, Eastern Cape Province, South Africa. Infect. Drug Resist..

[114] Zhou Y., Zhu X., Hou H., Lu Y., Yu J., Mao L., Mao L., Sun Z. (2018). Characteristics of diarrheagenic Escherichia coli among children under 5 years of age with acute diarrhea: A hospital based study. BMC Infect. Dis..

[115] Dionisio F., Domingues C.P.F., Rebelo J.S., Monteiro F., Nogueira T. (2023). The Impact of Non-Pathogenic Bacteria on the Spread of Virulence and Resistance Genes. Int. J. Mol. Sci..

[116] Anas M., Lone S.A., Malik A., Ahmad J. (2025). Antimicrobial Resistance and Public Health Risks Associated with Staphylococci Isolated from Raw and Processed Meat Products. Foodborne Pathog. Dis..

[117] Abdel-Raheem S.M., Khodier S.M., Almathen F., Hanafy A.T., Abbas S.M., Al-Shami S.A., Al-Sultan S.I., Alfifi A., El-Tarabili R.M. (2024). Dissemination, virulence characteristic, antibiotic resistance determinants of emerging linezolid and vancomycin-resistant *Enterococcus* spp. in fish and crustacean. Int. J. Food Microbiol..

[118] Moghimi B., Ghobadi D.M., Shapouri R., Jalili M. (2023). Antibiotic resistance profile of indigenous *Streptococcus thermophilus* and *Lactobacillus bulgaricus* strains isolated from traditional yogurt. J. Food Qual..

[119] Obioha P.I., Anyogu A., Awamaria B., Ghoddusi H.B., Ouoba L.I.I. (2023). Antimicrobial Resistance of Lactic Acid Bacteria from *Nono*, a Naturally Fermented Milk Product. Antibiotics.

[120] Chajęcka-Wierzchowska W., Zadernowska A., Zarzecka U., Zakrzewski A., Gajewska J. (2019). Enterococci from ready-to-eat food—Horizontal gene transfer of antibiotic resistance genes and genotypic characterization by PCR melting profile. J. Sci. Food Agric..

[121] Fontana C., Patrone V., Lopez C.M., Morelli L., Rebecchi A. (2021). Incidence of Tetracycline and Erythromycin Resistance in Meat-Associated Bacteria: Impact of Different Livestock Management Strategies. Microorganisms.

[122] Kläui A., Bütikofer U., Naskova J., Wagner E., Marti E. (2024). Fresh produce as a reservoir of antimicrobial resistance genes: A case study of Switzerland. Sci. Total Environ..

[123] Sabala R.F., Fukuda A., Nakajima C., Suzuki Y., Usui M., Elhadidy M. (2024). Carbapenem and colistin-resistant hypervirulent Klebsiella pneumoniae: An emerging threat transcending the egyptian food chain. J. Infect. Public Health.

[124] Rao M., Teixeira J.S., Flint A., Tamber S. (2024). Hazard Characterization of Antibiotic-resistant Aeromonas spp. Isolated from Mussel and Oyster Shellstock Available for Retail Purchase in Canada. J. Food Prot..

[125] Furlan J.P.R., Lopes R., Ramos M.S., Rosa R.D.S., Dos Santos L.D.R., Stehling E.G. (2024). The detection of KPC-2, NDM-1, and VIM-2 carbapenemases in international clones isolated from fresh vegetables highlights an emerging food safety issue. Int. J. Food Microbiol..

[126] Fanelli F., Caputo L., Quintieri L. (2021). Phenotypic and genomic characterization of *Pseudomonas putida* ITEM 17297 spoiler of fresh vegetables: Focus on biofilm and antibiotic resistance interaction. Curr. Res. Food Sci..

